# Reorganization of an Emergency Medical System in a Mixed Urban-Rural Area

**DOI:** 10.3390/ijerph191912369

**Published:** 2022-09-28

**Authors:** L’udmila Jánošíková, Peter Jankovič, Marek Kvet, Gaston Ivanov, Jakub Holod, Imrich Berta

**Affiliations:** 1 Faculty of Management Science and Informatics, University of Žilina, Univerzitná 1, 010 26 Žilina, Slovakia; 2 EMS Command and Control Centre of the Slovak Republic, Trnavská Cesta 8/A, 820 05 Bratislava, Slovakia

**Keywords:** emergency medical service, ambulance location, response time, coverage, computer simulation

## Abstract

The reorganization of an emergency medical system means that we look for new locations of ambulance stations with the aim of improving the accessibility of the service. We applied two tools that are well known in the operations research community, namely mathematical programming, and computer simulation. Using the hierarchical *pq*-median model, we proposed optimal locations of the stations throughout the country and within large towns. Several solutions have been calculated that differ in the number of stations that are supposed to be relocated to new positions. The locations proposed by the mathematical programming model were evaluated via computer simulation. The approach was demonstrated under the conditions of the Slovak Republic using real historical data on ambulance dispatches. We have concluded that (i) the distribution of the stations proposed by the hierarchical *pq*-median model overcomes the current distribution; the performance of the system has significantly improved even if only 10% of the stations are relocated to new municipalities; (ii) the variant that relocates 40% of the stations is a reasonable compromise between the benefits and induced costs; (iii) optimizing station locations in big towns can significantly improve the local as well as the nationwide performance indicators; the response times in two regional capitals has reduced by more than 4 min.

## 1. Introduction

Emergency medical services (EMS) are established to provide first aid to people who are in emergency situations due to injuries, attacks, or a sudden worsening of their medical conditions. In case of serious illness or injuries, a rescue team should reach the patient as soon as possible in order to minimize the physical or mental damage and to increase the chances of recovery. The responsiveness of the EMS system depends on a number of factors. From the viewpoint of the system, they can be classified as exogenous or endogenous. The exogenous factors are, for example, the quality of the road network, traffic density, or the weather. The endogenous factors are related to the management of the system. They include the amount, equipment and deployment of the ambulances, the skills of the staff, and the dispatch process. In this paper, we focus on the deployment of base stations where idle ambulances and their crews remain waiting for the next rescue dispatch.

We deal with an existing system with a fixed number of ambulances that provide services in a large-scale mixed urban-rural area. We want to improve the performance of the system, yet we also aim to avoid the massive investments associated with increasing the number of EMS units (ambulances and staff). We prefer to apply less expensive measures, specifically the re-deployment of the stations so that they are closer to potential patients. We base our solution on the current situation in Slovakia, where the distribution of the stations throughout the country is defined by government regulations. This means that the government specifies the number and types of ambulances, as well as their deployment within municipalities. Only one ambulance is allocated to every station. There may be multiple stations in big towns. Commercial and state providers apply for licenses to operate the individual stations. A provider with a license then finds a suitable building with a garage for the vehicle, and an office for the staff. If a provider holds multiple licenses in a town, then these stations can share the same building.

The day-to-day management of health-care provision is delegated to eight regional dispatch centers. Dispatch centers receive telephone requests for ambulance services, provide medical advice on the phone and organize the coordinated dispatch of appropriate resources (i.e., vehicles and personnel). In case of an emergency, callers have several options. They can either call the universal 112 number that is common in most EU countries or call a specific number (150—fire department, 155—EMS, 158—police). In cases where they call 112, the call-taker assesses the situation and either dispatches units or forwards the call to one of the three numbers above. Apart from Bratislava, these emergency dispatch centers share the location but are independent from each other regarding processes and organization.

The Slovak EMS system works in a Franco-German style. This means that the crew is qualified to provide medical treatment on the scene of an incident. After the initial treatment, the patient is either left at home or transported to an appropriate hospital for further treatment. There are two types of ambulances that differ in the equipment and qualification of the crew. Well-equipped advanced life support (ALS) units are staffed by a physician, a paramedic, and a driver. ALS ambulances are dispatched in cases of severe incidents, such as cardiac arrests, at which a patient’s condition requires an immediate and specialized intervention. Basic life support (BLS) ambulances are staffed by two paramedics. They provide first aid in non-life-threatening cases. After completing nursing school, EMS call-takers and paramedics have to complete either specialized secondary education or a bachelor’s degree in urgent care. EMS call-takers must complete a one-year specialized command and control centre course. Although we use the term paramedic, what the position entails falls somewhere between an emergency medical technician (EMT) and a paramedic in English-speaking countries. They undergo longer education and training than EMTs but do not have the competences a paramedic has in, for example, the UK (e.g., invasive airway intubation). EMS physicians have a medical degree and finish their residency (usually surgical or intensive care). Drivers undergo basic medical training (not with the purpose of treating patients but to assist paramedics or physicians).

Currently, there are 274 stations distributed within the country; 86 stations are of ALS type and the remaining 188 stations house a BLS ambulance. BLS ambulances transfer 71% of their patients to hospitals, while only approximately 53% of patients attended by ALS ambulances need to be transported to hospitals. ALS and BLS ambulances are mainly engaged in so-called primary interventions elicited by emergency calls. An additional 47 ambulances perform only secondary transfers of patients among hospitals.

Emergency call-takers assign one out of three priorities to each call during telephone triage: (1) the highest priority (the Slovak abbreviation is K) is assigned to patients who evidently are in life-threatening conditions and are likely to die without an immediate doctor’s intervention; (2) the N priority is assigned to patients with urgent and potential life-threatening conditions; and (3) the lowest priority M belongs to patients in non-life-threatening conditions. The dispatching strategy is as follows: (1) in the case of a priority K call, the closest ambulance available to the emergency site is always dispatched, regardless of its type; if it is a BLS ambulance, then the closest ALS ambulance is dispatched concurrently; (2) in the case of a priority N call, the closest BLS ambulance is dispatched, and after the patient’s condition has been evaluated, the crew may require a supporting ALS ambulance; (3) for the remaining calls, the closest BLS ambulance is dispatched, and only if the destination is too far for them, an ALS ambulance can respond to the call.

The station placement was set up in 2006. Since then, only marginal modifications have been made. However, the socio-demographic development of the population has resulted in changes in the demand for EMS. It seems that the system infrastructure does not correspond to the current demand because performance measures have been worsening (e.g., the average response time of BLS ambulances has increased by 2 min since 2010) [1,2]. The response time is regarded as the main performance measure. In Slovakia, it is defined as the time interval between the assignment of an ambulance by the dispatch centre and its arrival at the scene.

The network of the stations was designed empirically, without using any sophisticated decision support tool (mathematical modelling or computer simulation). However, plenty of mathematical models for ambulance location have been reported in the scientific literature [3,4,5]. The models more or less reflect the performance targets of the EMS systems. In addition to the response time, a common target is to reach a given percentage of patients within a pre-defined time limit. Mathematical programming models reflect the reality with a high level of abstraction. Because they neglect or strongly simplify the stochastic character of the EMS system, their objective function value is only a rough estimate of the corresponding performance indicator. A more precise image of the system performance can be obtained by using a detailed computer simulation model. Moreover, in contrast to a mathematical programming model, computer simulation allows for the evaluation of multiple performance criteria, such as the response time, the number of calls responded to within different time limits, the pre-hospital time, the vehicle utilization rate, or the total mileage. The paper [6] is an excellent review of the simulation models applied to emergency medical service operations. Most studies reviewed in [6], as well as those published later, e.g., [7,8], report the simulation experiments within the framework of a what-if analysis, without any connection to an optimization tool. However, the rapid development of the power of computing, the development of high-performance mathematical programming solvers, and the increasing availability of high-quality data have enabled the creation of efficient optimization procedures and detailed simulation models used for the evaluation of the deployment scenarios [9,10,11,12]. In this study, we join this research stream.

The aim of our research is to find out whether the stations can be re-distributed in a more efficient way, not only at the nationwide level but also at the city level. More specifically, our research questions are as follows:

1. What performance improvements are possible by optimizing station locations at the nationwide level?
2. What type of ambulances should be allocated to the stations?
3. How many stations need to be relocated to achieve a balance between significant performance improvement and the costs associated with the reorganization?
4. What additional improvement can be achieved by repositioning stations in big towns (regional capitals)?

## 2. Materials and Methods

In this study, we address the location problem in a large-scale area. Slovakia has 5.4 million inhabitants, and an area of 49.034 km^2^. The EMS system has to meet the demands in both urban and rural areas. The question remains how to model the spatial distribution of the demands over such a mixed territory. In the relevant literature two approaches are used: the demand zones are identical to either the postal code areas [10,13], or the elements of a rectangular grid [14,15]. However, neither of these two methods is ideal for a diversified region with an uneven density of population and road network. Therefore, this study proposes a new two-level approach to modelling the spatial distribution of EMS demand, and to finding the optimal deployment of stations in a large-scale mixed urban-rural area. At the upper level, the decisions made by the government are modelled. This means the optimal distribution of stations throughout the country is calculated. The solution specifies which municipalities will have one or multiple stations. At the lower level, further optimization is performed for individual municipalities. At this level, the municipality’s area is divided by a square grid. The grid cells represent demand zones. Preliminary experiments proved that a reasonable aggregation level of demand could be achieved with the cell size of 250 × 250 m. The demand points, as well as the candidate locations, are the nodes on the road network that are the nearest ones to the centers of the cells.

The demand at both levels is derived from the historical emergency calls of priority K and N (life-threatening and urgent conditions) because they are of primary concern to EMS. We do not consider the demand elasticity, i.e., the possibility that the demand of EMS could depend on the supply of the EMS service. The reason is that the accessibility of prehospital care is decreasing due to the decreasing number of GPs, so EMS remains the first choice to obtain professional first aid even in areas where ambulance response times are longer.

In this study, we endeavor to improve the infrastructure of the EMS system without increasing the number of EMS units. Therefore, we preserve the current number of ALS and BLS stations, but aim to find better locations for them. We suppose that the number of ambulances on duty is fixed during the course of a day, in contrast to many EMS systems where the number of units is lower during the night or at other quiet times. However, in Slovakia, this principle of having a flexible number and position of ambulances depending upon demand is not adopted. We do not exclude its application in the future, but it was not an issue in this research.

### 2.1. The Hierarchical pq-Median Model

The EMS system with two types of ambulance is a hierarchical system of interacting facilities of different types. According to the classification proposed in [16], the EMS system is a nested hierarchical facility system because a higher-level facility (ALS ambulance) provides all the services provided by a lower-level facility (BLS ambulance), and some additional services. As far as the spatial configuration is concerned, we deal with a non-coherent system because the demand zones that are assigned to a particular BLS station need not be assigned to the same ALS station. If the objective is to minimize the demand weighted total distance (or travel time, respectively) between customers and facilities, then we face a median-type system. In addition, if the number of facilities of each type are given in advance, then the system can be described as a hierarchical *pq*-median problem. The paper [17] introduced the *pq*-median model for coherent systems. In our previous study [12], we proposed the modification of this model for the non-coherent EMS system. In the following paragraphs, we will present the mathematical programming formulation of the problem and analyze the properties of the model and their consequences for the data pre-processing and the interpretation of the results.

We will use the following notation: let *I* be the set of candidate locations and *J* be the set of demand points. Let *t_ij_* stand for the shortest travel time from the candidate location *i* ∈ *I* to the demand point *j* ∈ *J*. The volume of demand in demand zone *j* will be denoted by *b_j_*.

At the lower hierarchical level, we look for optimal locations of all *p* stations regardless of their type. This is modelled by the decision variable *y_i_*, which takes the value of 1 if a station is located at node *i*, otherwise *y_i_* = 0. To calculate the average travel time from a station to an incident site, we need to create the catchment areas of the stations, i.e., to assign demand zones to the located stations. The decision variable *x_ij_* ∈ {0, 1} captures the assignment of demand zone *j* to a station located at node *i*. Using this notation, the model can be written as follows:

(1) $$\mathrm{minimize}\sum_{i\in I}\sum_{j\in J}t_{ij}b_{j}x_{ij}.$$

(2) $$\mathrm{subject}\mathrm{to}\sum_{i\in I}x_{ij}=1\;\;\mathrm{for}\;j\;\in \;J$$

(3) $$x_{ij}\leq y_{i}\;\;\;\mathrm{for}\;i\in \;I,\;j\in \;J$$

(4) $$\sum_{i\in I}y_{i}=p$$

(5) $$x_{ij},\;y_{i}\in \left\{0,1\right\}\;\;\;\mathrm{for}\;i\;\in \;I,\;j\in \;J$$

The objective function (1) minimizes the demand weighted total travel time from the stations to the demand points. The average travel time is calculated by dividing the objective function value by the total volume of demand $\sum_{j\in J}b_{j}$. Equations (2) ensure that every demand zone will belong to the catchment area of exactly one station. Equations (3) allow demand zone *j* to be assigned to candidate location *i* only if a station is opened there. Equation (4) allows exactly *p* stations to be opened. The remaining Equations (5) are the integrality restrictions on decision variables.

At the higher hierarchical level, we select *q* locations among the optimal locations of the *p* stations specified at the lower level and allocate ALS ambulances to them. The remaining *p*–*q* stations will then house BLS ambulances. The optimization criterion is again the total travel time between the ALS stations and the demand points. Decision variables *u_i_* ∈ {0, 1} indicate whether an ALS station is located at node *i*. The catchment area of an ALS station located at node *i* is defined by assignment variables *v_ij_* ∈ {0, 1}. The upper level of the system is then described by the following model:

(6) $$\mathrm{minimize}\sum_{i\in I}\sum_{j\in J}t_{ij}b_{j}v_{ij}$$

(7) $$\mathrm{subject}\mathrm{to}\sum_{i\in I}v_{ij}=1\;\;\;\mathrm{for}\;j\;\in \;J$$

(8) $$v_{ij}\leq u_{i}\;\;\;\mathrm{for}\;i\;\in \;I,\;j\in \;J$$

(9) $$u_{i}\leq y_{i}\;\;\;\mathrm{for}\;i\;\in \;I$$

(10) $$\sum_{i\in I}u_{i}=q\;$$

(11) $$u_{i},\;v_{ij}\in \left\{0,1\right\}\;\;\;\mathrm{for}\;i\;\in \;I,\;j\in \;J$$

The objective function (6) minimizes the total travel time needed by ALS ambulances to reach all patients. Constraints (7) assign every demand zone *j* to the catchment area of exactly one ALS station *i*. Constraints (8) say that a demand zone *j* can be assigned only to an open ALS station. Constraints (9) allow an ALS ambulance to be allocated only to a station located at node *i* at the lower level of hierarchy (optimal values of variables *y_i_* are regarded as coefficients in this model). Constraint (10) limits the number of located ALS stations to their current number *q*. The remaining constraints (11) specify the binary variables.

The model was solved using the optimization software FICO XPRESS 8.0 (64-bit, release 2016).

### 2.2. Practical Implications

The model is based on a few simplifying assumptions. Most importantly, it is assumed that a station is available to respond to a call immediately, and its capacity is unlimited. That is why the model always assigns calls to the closest station (at both levels of hierarchy). Another consequence of this assumption is that it is pointless to locate multiple stations at the same candidate location *i* (the second station at the same node *i* does not decrease the objective function value). This would lead to a practically infeasible solution at the nationwide level because the catchment areas of some stations located in highly populated municipalities would be enormous, their workload would be extreme, and, as a result, the response times would grow above an acceptable value. Therefore, we have to analyze the current distribution of the stations and the population distribution and fix one or more stations in big towns before the optimization.

To obtain more realistic results from the optimization model, we apply two rules that fix some stations at their current positions.

The first rule ensures that backup facilities are available in high-demand areas. We look at the current distribution of the stations and preserve one or more stations in towns where they are supposed to be fully charged. We set the capacity of a station to the average population falling on one station. If the population of a town exceeds the capacity of the stations currently sitting in the town, we leave the stations unmoved. Otherwise, we fix necessary stations (by dividing the population by the capacity) and allow the remaining stations to be relocated. Consequently, we reduce the demand in the town by the demand assigned to the fixed stations.

The second rule is applied to small towns where two stations are in operation today, although the population in the town is below the capacity of a station. The reasons for operating multiple stations in small municipalities are not apparent to us (maybe a good hospital is nearby) but we respect these managerial decisions from the past (to some extent), and we fix one station in the town. The other station becomes free, and it can be relocated elsewhere.

At the upper level of the hierarchy, we specify the types of the stations. The optimization model (6)–(11) decides which of the fixed stations and the stations located in municipalities by the model (1)–(5) will house an ALS ambulance. If the fixed stations are of an ALS type, then their type will not change. However, the fixed BLS stations can be switched for ALS stations. The types of all other stations can be changed.

Thus, the output of the nationwide optimization is a list of municipalities and the number of ALS and BLS stations that are sited in every municipality. Then, the second stage of the optimization takes its turn. Here, the optimal distribution of the stations inside the towns is to be calculated. The deployment of the stations can significantly affect the response time, especially in large towns. It is supposed that the service availability will be higher if the ambulances are scattered throughout the territory, rather than concentrated in one place (as it is usual in Slovak cities). As was mentioned above, the demand zones in towns are created by using a rectangular grid. The size of the grid is a compromise between the computability of the model and the spatial resolution that adequately represents the distribution of the demand, and it accurately specifies the locations of the stations. Our experience concerns only middle-sized towns to 500,000 inhabitants. For such towns, we can recommend a grid resolution of 250 m.

### 2.3. Computer Simulation Model

**Figure 1:**
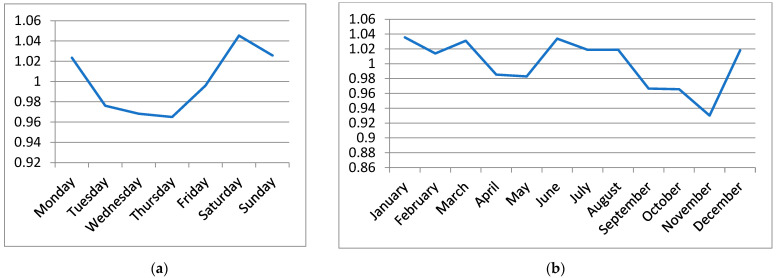
(**a**) The ratio of the number of trips on individual days to the daily average in 2019; (**b**) The ratio of the number of trips in individual months to the monthly average in 2019.

**Figure 2:**
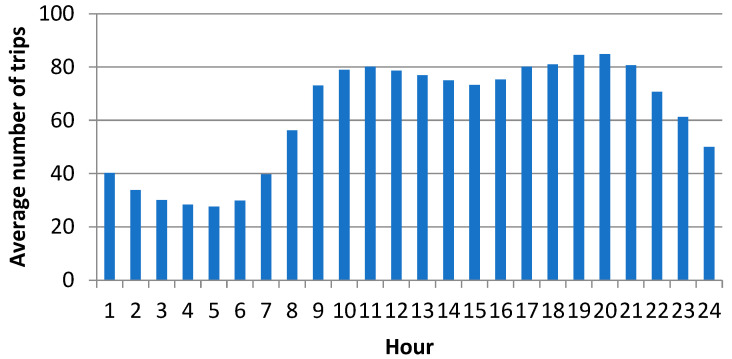
Trip rate by time of day, 2019.

We have built a detailed simulation model in Java language [11] and calibrated it by using the actual data on EMS trips gathered by the EMS Command and Control Centre of the Slovak Republic. The data covers the period from January to September 2020. The COVID-19 pandemic was not very intensive at that time, yet, and its influence on the performance of the EMS system was only negligible. In this research, we worked with depersonalized data on the EMS trips. Each record of a trip contains time stamps, the priority that the call-taker has assigned to the patient, the patient’s diagnosis, the destination hospital, and information on the ambulance. The data have been used to describe the time and spatial distribution of the emergency cases. We have observed that the number of trips was almost invariant regarding the day of the week and month of the year (Figure 1). The call arrival rate changed during the day with the morning peak between 9 and 11 a.m. and the evening peak between 6 and 9 p.m. (Figure 2). Therefore, the simulated arrival of calls follows a non-homogeneous Poisson process, with the rate specific for each hour of the day.

The data allowed us to derive not only the time distribution but also the spatial distribution of patients. The monthly number of EMS patients for every region in Slovakia was calculated. The precise positions of the simulated calls within a region were specified by using the LandScan database (https://landscan.ornl.gov, accessed on 18 December 2021). LandScan data represents an ambient population distribution; it means the average presence of people during a day. The advantage of such a model is that it reflects the daily activity of people, so it takes into account not only the permanent residents but also the commuters who travel there for work or to schools. A grid cell corresponds to an area of 30″ × 30″ (arc-seconds) in the WGS84 geographical coordinate system. The territory of the Slovak Republic is covered by 70,324 grid elements.

The spatial distribution of patients is modelled in the following manner: the call that has been generated by the Poisson process is assigned first to a region and then to a grid cell with a probability that is proportional to its population. Inside the grid cell, the call is assigned randomly to a node on the road network.

The historical data was also used to model the age of the patients, their priority, and diagnosis. Calls of all priorities (immediately life-threatening, urgent and non-life-threatening) are simulated. A patient’s condition affects the time the rescue team spends on-site, as well as the selection of the destination hospital. We differentiate children’s hospitals and hospitals that admit only adults. Moreover, there are specialized hospitals that provide specific care for patients with particular diagnoses, e.g., heart diseases. The on-scene time is modelled using a probability distribution that depends on the patient’s diagnosis and crew’s qualification. The average on-scene time ranges approximately between 20 and 30 min, and in general it is higher for a BLS ambulance than for an ALS ambulance. The probability of a subsequent transfer to hospital is different with each ambulance type. BLS ambulances transfer approximately 71% of their patients to hospital, while ALS ambulances only 53%. In hospital, the rescue team hand over the patient to hospital staff, then they may spend some time cleaning and resupplying the vehicle. The time needed to perform these tasks is called drop-off time. The probability distribution of the drop-off time is modelled separately for every hospital. In most cases, the Erlang distribution fits well. The average drop-off time ranges from 14 to 38 min.

A patient’s priority has an impact on the ambulance dispatch and travel speed. The movement of ambulances is based on a digital road network that was downloaded from the OpenStreetMap database (https://www.openstreetmap.org, accessed on 16 April 2019). It is assumed that an ambulance always travels along the route with the shortest time. The speed of ambulances depends on the priority of the call, the road category, and time of day. It is lower in rush hours (from 6:30 to 9 a.m. and from 3 to 6 p.m.) and within built-up areas. The ambulance can be dispatched to the next call after finishing the service on the scene or in the hospital. In this aspect, our simulation model adequately reflects reality because an ambulance can be re-dispatched while it is en route to its base station.

Such detailed modelling of on-scene times, drop-off times and the movement of ambulances is unique and does not occur in any simulation study published in the literature. The model was thoroughly verified and validated, using three methods:

- Animation during the simulation run;
- Consultations with medical experts;
- Comparing the average response times to actual statistics from the current system (Table 1).

The model gives slightly more optimistic results in comparison to the real system because some processes had to be neglected due to lack of reliable data, e.g., technical breaks of vehicles. However, the differences are so small that we can consider the model valid.

The simulation model allows for the evaluation of all performance indicators that are monitored by the EMS authorities. We focus on the following indicators:

1. Average response time evaluated separately for every patient´s priority and in common for all patients;
2. Percentage of calls responded to within 15 min, since a 15 min response time is regarded as standard in Slovakia;
3. Average response time to the patients who have the most severe diagnoses; the most severe conditions are denoted as the First Hour Quintet (FHQ) and include: chest pain, severe trauma, stroke, severe respiratory difficulties, and cardiac arrest [18];
4. Percentage of FHQ calls responded to within 8 min;
5. Average ambulance workload;
6. Total mileage of ambulances.

The fourth indicator is used to compare the design to other European countries where the 8-min response time is a widely accepted standard. The European Emergency Data (EED) Project [18] defines the percentage of the highest priority calls responded to within 8 min as one of five key indicators of the EMS performance. The project evaluated the EMS services in 11 EU Member States. The indicator ranged from 52% in Finland to 89% in Denmark with an average of 66.9%.

## 3. Results

### 3.1. Parameter Setting

The demand model for infrastructure optimization included calls of priority K and N (life-threatening and urgent) responded to from August 2018 to September 2021. The total number of these calls was 532,115.

Currently, there are 274 stations in Slovakia. At the nationwide level, we applied the rules specified in Section 2.2, and we fixed 76 stations at their current positions. The remaining 198 stations were subject to optimization.

At the lower level of the hierarchical model, where the type of a station does not play a role, the potential locations for the stations coincide with the locations of the demand nodes, i.e., *I* = *J*. This assumption holds for the country, as well as for the cities. The cardinalities of the sets for the nationwide optimization are |*I*| = |*J*| = 2934. In the cities, the cardinalities of the sets depend on the size of the city.

One simulation run took 91 days of the system operation. For every set of station locations, 10 replications were performed. The average results of 10 replications are reported in the following tables.

### 3.2. Experiments with a Different Number of Relocated Stations

In the first set of experiments, we evaluated the different scope of the proposed changes in the infrastructure. We performed the optimization with regard to the whole country, omitting the optimization within municipalities. We experimented with different numbers of stations that could be relocated. We evaluated six scenarios. The number of relocated stations ranged from 10 to 50% of the current number, and finally it was not restricted at all, i.e., 27, 55, 82, 110, 137, and 198 stations were allowed to change their positions. The solution of the optimization model was post-processed this way: the stations that were not relocated by the model remained at their current addresses. The other stations were placed at the central nodes of the municipalities.

The static evaluation of the model solutions is given in Table 2. The average travel times and coverage are the optimistic estimations of real values due to the simplifications present in the model. In the last scenario, where the number of relocated stations was not constrained, the model placed 151 stations (55% of all stations) into new municipalities.

**Figure 3:**
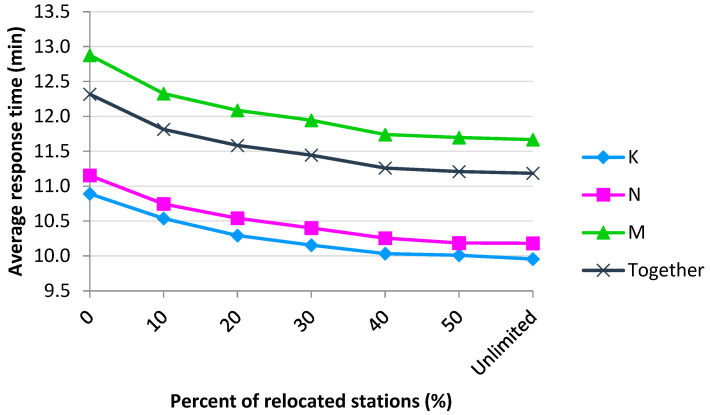
The average response time decreases with increasing level of system optimization.

The results of the simulation experiments are given in Table 3. We can see that even the most subtle modification of the system that relocates 10% of stations induces a statistically significant reduction in the average response time for all groups of patients in comparison to the current distribution of the stations (September 2021). In addition, the scenarios with 20, 30 and 40% of relocated stations significantly reduce all response times in mutual comparison to the previous configuration with less severe infrastructure modification (with one exception of priority K for the 40% scenario). When the number of relocated stations increases even more, the response time to the most critical FHQ patients does not significantly decrease. The trends in the reduction in response times can be observed in Figure 3.

The coverage of patients within the 15-min standard continuously increases from the current value of 70.61% to 76.99%. The same trend can be observed in the 8-min coverage of the patients with a FHQ diagnosis, which increases from 34.20% to 41.30%. In the last scenario, where the number of relocated stations is not restricted, the average response time to the FHQ patients is 1 min shorter than in the current situation, which constitutes a substantial improvement. If we project this reduction to the survival probability of cardiac arrest patients according to the function published in [11], we come to the conclusion that up to 68 more people could be saved annually just in this diagnosis group. For all people whose life is in danger, any reduction in response time alleviates the consequences of the accident.

An ambulance workload is almost identical in all scenarios. In general, the workload of BLS ambulances is one percentage point higher than that of ALS ambulances.

The total mileage in all scenarios is lower than it is nowadays. The length of ambulance routes is the least in the 30% scenario (by 91,859 km shorter than at present, which means an annual saving of 367,436 km). The ambulance routes become longer in the last three scenarios, where at least 40% of stations are relocated. The reason is that the stations are closer to the patients but more distant from the hospitals.

The results of the simulation study suggest that the relocation of 50% of stations (and more) would bring only a marginal improvement in the service availability in comparison to less drastic changes. The managerial opinion is that the costs associated with the relocation of so many stations would not be balanced by the insignificant improvements in response times and coverage. The last scenario, with unlimited redeployment, increases the coverage of both thresholds only by 0.5% in comparison to the 40% scenario. In addition, the transportation costs increase with the extent of the reconstruction. Therefore, we recommend the mild reorganization of the system infrastructure that relocates 40% of stations.

**Figure 4:**
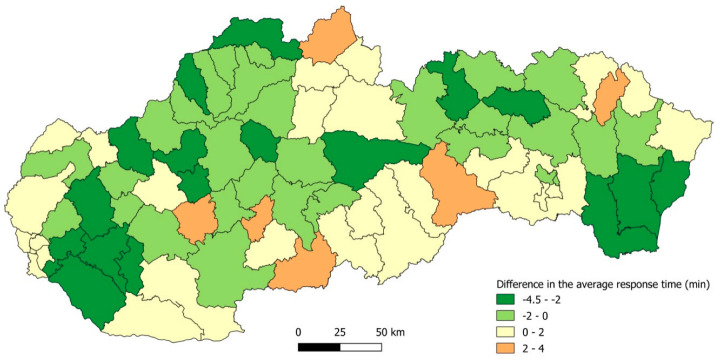
Difference in the average response time to the patients of priority K and N. A comparison of the 40% scenario with the current deployment of stations.

Figure 4 offers another view of the impact of the stations’ redeployment. The alteration in the average response time to the patients of priority K and N is displayed for 79 districts in Slovakia. The 40% scenario, in which 110 stations change their position, is compared to the current deployment of the stations. This moderate system reorganization reduces the average response time in 46 districts; in 17 of them by more than 2 min. However, it must be said that this improvement is at the expense of worsening the average response time in the remaining 33 districts, in 6 of them by more than 2 min.

### 3.3. Optimal Locations of Stations in Towns

In the second set of experiments, we looked for the answer to the fourth research question: what additional improvement could be achieved by repositioning the stations in big towns (regional capitals)? We applied the model (1)−(11) to eight regional capitals in Slovakia. We supposed that the distribution of the stations throughout the country had been calculated at the previous stage of the optimization process. The optimal number and types of stations in the towns were the input parameters for this second stage. We used the results of the 40% scenario that was recommended as a reasonable nationwide solution. Table 4 lists the regional capitals, including their population and the number and types of stations, before and after the nationwide optimization. We can see that the optimization model placed 11 BLS stations outside the regional capitals.

When optimizing the locations in the towns, we did not restrict the number of relocated stations at all. As a result, all stations in the towns changed their current positions.

The optimal locations of the stations in the regional capitals were combined with the optimal distribution of the stations throughout the country, calculated for the 40% scenario, and evaluated using computer simulation. The simulation experiments have revealed that the optimization of the locations in the regional capitals improves the service not only in the towns themselves, but it has an impact on the whole country, too. Table 5 compares the results with and without the optimization of the regional capitals. We can see that the reduction of all response times is statistically significant, and at the same time the coverage in both limits is better.

The simulated performance indicators for the individual regional capitals are given in Table 6 and Table 7. The values of the indicators for the current state and for the optimal deployment without and with the optimization in the towns are reported. The fourth column for each indicator gives the difference made by the 40% scenario combined with the optimization in the towns with respect to the current distribution of the stations. The capital, Bratislava, and the second largest Slovak town, Košice, are assessed at the district levels.

The EMS simulation with the current distribution of the stations has revealed that there are big differences in service availability among the towns. The best service is provided in the district of Košice I. Here, the average response time is 6.13 min, and 80.59% of FHQ calls are responded to within 8 min. The worst availability is in Trnava, where the average response time is 13.15 min to all patients, regardless of their priority, and the percentage of FHQ calls responded to within 8 min is only 15.82%. The situation in Banská Bystrica is only slightly better; the average response time is 11.10 min, and the 8-min coverage is 16.15%. At present, in both towns, all ambulances are housed at the same address, located outside the most populated town sections.

**Figure 5:**
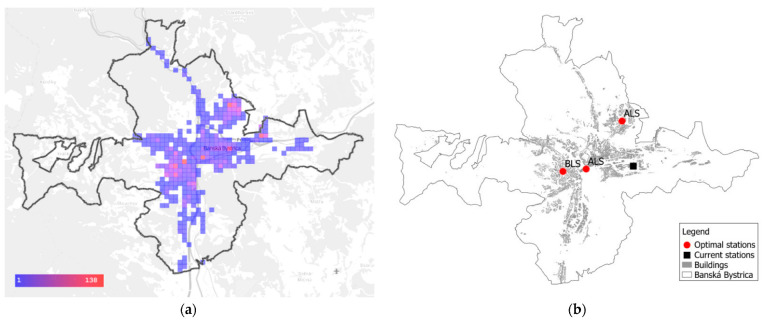
City of Banská Bystrica. (**a**) Distribution of demand; (**b**) Current and optimal locations of the stations; currently 4 stations are at the same address.

The optimization diminishes the differences among the towns, and it results in a fairer distribution of the stations. The quality of service improves in most regional capitals in comparison to the current state, as well as with the optimal deployment where the stations in the towns remain at their current addresses. The most significant improvement is observed in the two currently worst towns: Trnava and Banská Bystrica. The response times are reduced by more than 5 min in Trnava, and by more than 4 min in Banská Bystrica. The result in Banská Bystrica is remarkable because the model removes one station from the town. Figure 5 illustrates the situation in Banská Bystrica. The distribution of the demand is given in panel (a), and the current and optimal locations of the stations are given in panel (b).

In the capital, Bratislava, the best improvement can be seen in the district of Bratislava III. The response times in the district become shorter by approximately 2 min, although the optimal design locates four fewer stations there. Apparently, today, the stations are located inconveniently, and they provide backup service for the other parts of the city and suburbs. On the other hand, the response times become worse by 2 min in the district of Bratislava V. Because we do not fix the stations in the towns, the model (1)−(11) distributes the stations throughout the town area, and it does not place more than one station at the same node. This seems to be problematic with highly populated housing estates, such as Petržalka in the district of Bratislava V, where more than 100,000 people live. Currently, there are five stations there, but the model places only two stations there. Consequently, the workload of the stations is extremely high (49.2 and 54.6%) and, often, ambulances from more distant stations have to rescue patients in the district.

The quality of service in Košice is approximately the same as before the optimization, although the number of stations is lower by three.

After the optimization of regional capitals, we evaluated the service accessibility separately for urban and rural areas. We found that optimization improves the response time balance between urban and rural areas. Currently, the average response time in urban and rural areas is 11.02 min and 12.47 min, respectively, meaning the difference of 87 s. The optimal design reduces the difference to 22 s with the average response times of 10.75 min and 11.12 min, respectively. This result also suggests that the current network of stations is not perfect and can be improved to increase the positive perception of the service by the population.

## 4. Discussion

We have been asked to perform this analysis by the EMS Commands and Control Centre of the Slovak Republic in order to support their decision-making process related to investment and reforms specified in the Next Generation EU: Pandemic Recovery Plan to a build greener, more innovative, and stronger Europe. The goal was to foresee possible improvements in terms of performance indicators rather than to propose a practical solution suitable for direct implementation. The outputs of the optimization are supposed to support decisions made by responsible authorities. Decision-makers have to assess the proposed changes in the infrastructure carefully by taking into account practical aspects that the mathematical model omits, e.g., the quality of the road network in the neighborhood of new stations. Further, they have to consider whether accessibility deterioration in some regions would be acceptable or if additional resources are needed. One of the practical issues neglected in the urban model is that a station can be placed at any central node of the demand grid. Therefore, the solution of the model should be interpreted in terms of the wards surrounding the calculated optimal station location rather than the station´s precise address. In the future, potential sites for station locations should be specified, accounting for practical issues, such as the existence of a suitable facility, its tenancy costs, connection to the road network, etc. Then, the set of candidate locations will contain genuine potential sites.

We are aware of several other limitations of our research. We do not take into account the costs of closing the existing stations and of opening the new ones. We were supposed to calculate an optimistic estimation of the improvement that would result from the redeployment. We note that, in practice, it may not be acceptable to relocate all the proposed stations. However, the proposed model can easily cope with such a limitation by fixing the desired stations in their current positions, and by modifying the set *I* and the *p* and *q* parameters appropriately.

With regards to the mathematical programming model, in our previous studies we experimented with several other models, using response time objectives [11,12]. None of these models outperformed the hierarchical *pq*-median model. Several other studies demonstrate successful applications of location models that minimize the average response time. For example, Sasaki et al. [19] determined the optimal ambulance locations using the current and predicted EMS demand in the city of Niigata, Japan. Dzator and Dzator [20] applied the *p*-median model to locate ambulance stations in two sub-regions of the Perth Metropolitan area, Australia. Toro-Díaz et al. [21] compared several objectives using two case studies—the city of Edmonton, Canada, and Mecklenburg County, USA—and came to the conclusion that, “the solution minimizing response time is overall better than the one maximizing coverage”. In addition, the models that maximize the expected number of survivors (e.g., [22]) actually minimize the response time because the probability of survival is a non-increasing function of the response time.

The study [23] compared several models for EMS station location in a city. The modular capacitated location model outperformed other models. However, in this research, we have found that the hierarchical *pq*-median model produces the same results as the modular capacitated location model does, but it consumes much less computing time.

The performance of the optimized system infrastructure can be evaluated from different points of view by using computer simulation. A detailed simulation model of an EMS system is used by Strauss et al. [7] to analyze the consequences of amended parameters, such as the number of EMS units, relocation of EMS stations, and the use of multi-copters. However, this discrete event simulation (DES) model has some drawbacks in comparison to our agent-based model, e.g., it is difficult to dispatch the ambulance when it is driving from the hospital back to the station. Therefore, in the study [7], all vehicles start their trips at base locations. The DES study [10] overcomes this disadvantage by estimating the position of the ambulance at the moment, when it is being re-dispatched. The model [8] is similar to our simulation approach. This is a hybrid model where call-takers, ambulances, emergency doctors, and helicopters are modelled as agents, while emergency calls enter the system as events. The agent-based approach allows ambulances to track their rides so that their position is known when a new call arrives, which emulates the visualization of the ambulance’s position at a dispatch centre.

Probably the most important input parameter that affects not only the optimal locations but also the simulation results is the travel time along the road network. The travel time depends on the average speed of the ambulances. In turn, it depends on the traffic density during the course of a day, and on the road categories. In this research, we have employed rather old estimates from the study [11]. However, we hope that in the future we will be able to obtain up-to-date GPS traces of ambulance trips, and to derive more exact estimates, such as in [24].

The outputs of our study support the strategic as well as tactical decisions of the EMS managers and urgent health care providers. Our approach can be applied to any region with diverse geography, varied road network quality, and uneven population density, where a tiered EMS system with different types of vehicles operates. Such systems are common in many European countries, such as Germany, France, Greece, Switzerland, Austria, Czech Republic, Hungary, and Poland [7,25,26,27].

## 5. Conclusions

In this research, we have demonstrated a practical approach to the optimization of the EMS system infrastructure. Using a faithful computer simulation model, we have proved that a simple mathematical programming model combined with data pre-processing is able to design an efficient distribution of the EMS stations in a large-scale and diversified area. The main findings of our research are as follows:

1. The simple *pq*-median model can be used to find optimal locations for the stations at different levels of spatial resolution.
2. The *pq*-median model is able to specify optimal locations for emergency units of different types in a tiered EMS system.
3. The distribution of the stations proposed by the model overcomes the structure proposed by experts; the performance improves significantly, even if only 10% of stations are relocated to new municipalities.
4. The solution that relocates 40% of stations is a trade-off between the extent of changes and their positive impact on the service availability.
5. The optimization of the stations’ deployment in big towns can significantly improve the local as well as the nationwide performance indicators.

Our study is part of the source materials that will be submitted to the government to support their decision on the optimal EMS station network.

## Figures and Tables

**Table 1:** Actual and simulated average response times (ART) in Slovakia.

Patient’s Priority	Actual ART (min)	Simulated ART (min)
K	11.2	10.9
N	11.3	11.2
M	13.2	12.9

**Table 2:** Results of the optimization model.

	Current Locations (September 2021)	10% of Stations at New Positions	20% of Stations at New Positions	30% of Stations at New Positions	40% of Stations at New Positions	50% of Stations at New Positions	Unlimited Number of Stations at New Positions
The number of BLS stations switched to ALS stations		8	7	6	5	3	4
The number of ALS stations switched to BLS stations		8	8	8	7	5	3
The number of relocated stations		27	55	82	110	137	151
The number of relocated BLS stations		24	48	71	96	119	133
The number of relocated ALS stations		3	7	11	14	18	18
Average travel time from the closest station (min)	5.73	5.28	5.04	4.92	4.83	4.77	4.76
Average travel time from the closest ALS station (min)	7.98	7.75	7.73	7.73	7.73	7.73	7.74
Theoretical 15-min coverage (% population)	98.48	98.82	98.98	99.12	99.13	99.24	99.22

**Table 3:** Simulation results.

		Current Locations (September 2021)	10% of Stations at New Positions	20% of Stations at New Positions	30% of Stations at New Positions	40% of Stations at New Positions	50% of Stations at New Positions	Unlimited Number of Stations at New Positions
Average response time (min) 95% confidence interval	K	10.9 (10.83; 10.95)	10.5 (10.48; 10.60)	10.3 (10.27; 10.31)	10.2 (10.08; 10.23)	10.0 (9.98; 10.09)	10.0 (9.93; 10.09)	10.0 (9.92; 10.00)
	N	11.2 (11.12; 11.18)	10.7 (10.72; 10.77)	10.5 (10.51; 10.57)	10.4 (10.36; 10.44)	10.3 (10.23; 10.28)	10.2 (10.16; 10.21)	10.2 (10.16; 10.20)
	M	12.9 (12.86; 12.89)	12.3 (12.29; 12.36)	12.1 (12.07; 12.11)	11.9 (11.93; 11.96)	11.7 (11.72; 11.76)	11.7 (11.68; 11.72)	11.7 (11.64; 11.69)
	Together	12.3 (12.31; 12.33)	11.8 (11.79; 11.84)	11.6 (11.57; 11.60)	11.4 (11.43; 11.46)	11.3 (11.24; 11.28)	11.2 (11.19; 11.23)	11.2 (11.17; 11.20)
% of calls responded to within 15 min (%)		70.61	73.57	74.76	75.69	76.48	76.68	76.99
Average response time to FHQ patients (min)		11.9 (11.82; 11.89)	11.4 (11.32; 11.38)	11.2 (11.13; 11.19)	11.0 (10.96; 11.07)	10.8 (10.81; 10.88)	10.8 (10.75; 10.82)	10.8 (10.71; 10.81)
% of FHQ calls responded to within 8 min (%)		34.20	36.52	38.41	39.52	40.81	41.08	41.30
Average workload of BLS ambulances (%)		31.1	31.2	31.0	31.0	31.0	31.0	31.0
Average workload of ALS ambulances (%)		30.2	29.8	29.8	29.7	29.6	29.5	29.5
Total mileage of ambulances (km)		4,738,433	4,686,817	4,652,063	4,646,574	4,665,197	4,671,190	4,688,271

**Table 4:** Regional capitals in Slovakia.

Town	Population (December 2021)	Current State (September 2021)		40% of Stations at New Positions	
		BLS	ALS	BLS	ALS
Banská Bystrica	75,317	2	2	1	2
Bratislava	475,577	15	3	11	3
Košice	227,458	6	3	2	3
Nitra	77,610	2	2	2	2
Prešov	83,897	3	2	2	2
Trenčín	54,458	2	2	0	2
Trnava	63,194	1	1	2	1
Žilina	81,940	2	2	2	2
Total	1,139,451	33	17	22	17

**Table 5:** Simulation results for the 40% scenario with and without optimization in towns.

		Without Optimization in Towns	With Optimization in Regional Capitals
Average response time (min) 95% confidence interval	K	10.0 (9.98; 10.09)	9.7 (9.61; 9.72)
	N	10.3 (10.23; 10.28)	9.9 (9.88; 9.92)
	M	11.7 (11.72; 11.76)	11.6 (11.61; 11.66)
	Together	11.3 (11.24; 11.28)	11.1 (11.06; 11.08)
% of calls responded to within 15 min (%)		76.48	77.17
Average response time to FHQ patients (min)		10.8 (10.81; 10.88)	10.6 (10.56; 10.61)
% of FHQ calls responded to within 8 min (%)		40.81	42.54

**Table 6:** Performance indicators with respect to all patients regardless of their priority.

Area	Average Response Time (min)				% of Calls Responded to within 15 min			
	Current State	40% of Stations at New Positions	Optimization of Regional Capitals	Difference wrt Current State	Current State	40% of Stations at New Positions	Optimization of Regional Capitals	Difference wrt Current State
Banská Bystrica	11.10	8.86	7.06	−4.04	86.67	92.06	96.54	9.87
Nitra	8.73	7.89	6.83	−1.90	87.23	93.55	95.29	8.06
Prešov	6.36	5.20	4.80	−1.56	92.37	98.03	98.20	5.83
Trenčín	10.39	10.81	10.75	0.36	80.27	79.80	79.51	−0.76
Trnava	13.15	9.17	7.89	−5.26	71.12	86.98	91.09	19.97
Žilina	10.69	9.59	9.65	−1.04	82.94	90.27	87.38	4.44
Bratislava I	8.19	10.73	7.59	−0.60	95.63	87.79	93.45	−2.18
Bratislava II	10.00	10.36	8.86	−1.14	86.57	52.77	89.43	2.86
Bratislava III	13.60	15.66	11.68	−1.92	70.63	59.93	77.29	6.66
Bratislava IV	11.20	12.70	12.45	1.25	78.58	71.33	75.07	−3.51
Bratislava V	8.73	9.16	11.39	2.66	90.66	88.97	79.29	−11.37
Košice I	6.13	6.30	6.72	0.59	98.20	98.66	94.31	−3.89
Košice II	9.51	10.89	9.87	0.36	82.19	74.51	78.24	−3.95
Košice III	8.15	7.30	8.12	−0.03	93.58	95.55	89.51	−4.07
Košice IV	6.67	7.17	7.60	0.93	97.18	94.16	93.64	−3.54

**Table 7:** Performance indicators with respect to FHQ patients.

Area	Average Response Time (min)				% of Calls Responded to within 15 min			
	Current State	40% of Stations at New Positions	Optimization of Regional Capitals	Difference wrt Current State	Current State	40% of Stations at New Positions	Optimization of Regional Capitals	Difference wrt Current State
Banská Bystrica	11.31	9.32	6.63	−4.68	16.15	41.49	72.58	56.43
Nitra	8.20	7.66	6.44	−1.76	61.33	64.15	74.49	13.16
Prešov	6.02	5.05	4.44	−1.58	82.35	89.93	93.64	11.29
Trenčín	9.61	10.06	10.04	0.43	46.24	42.20	43.39	−2.85
Trnava	13.05	9.31	7.61	−5.44	15.82	47.20	63.42	47.60
Žilina	10.57	9.77	8.90	−1.67	32.53	36.59	47.75	15.22
Bratislava I	7.52	9.71	6.94	−0.58	64.98	44.13	75.06	10.08
Bratislava II	9.04	9.35	7.89	−1.15	55.40	53.75	66.07	10.67
Bratislava III	12.45	14.07	10.33	−2.12	32.61	23.35	47.47	14.86
Bratislava IV	10.27	11.46	10.81	0.54	49.28	41.81	41.76	−7.52
Bratislava V	8.05	8.33	10.08	2.03	65.05	63.91	51.94	−13.11
Košice I	5.74	5.88	5.89	0.15	80.59	82.03	78.85	−1.74
Košice II	9.23	10.45	9.42	0.19	59.48	50.49	54.31	−5.17
Košice III	8.06	7.14	7.32	−0.74	57.37	68.45	67.35	9.98
Košice IV	6.10	6.63	6.69	0.59	78.12	73.55	70.15	−7.97

## Data Availability

The datasets used and/or analyzed during the current study are available from the corresponding author upon reasonable request.
